# Matrix 3D ultrasound-assisted thyroid nodule volume estimation and radiofrequency ablation: a phantom study

**DOI:** 10.1186/s41747-021-00230-4

**Published:** 2021-07-29

**Authors:** T. Boers, S. J. Braak, M. Versluis, S. Manohar

**Affiliations:** 1 Multi-Modality Medical Imaging group, TechMed Centre, University of Twente, Enschede, The Netherlands; 2 Department of Radiology, Ziekenhuis Groep Twente, Almelo, The Netherlands; 3 Physics of Fluids group, TechMed Centre, University of Twente, Enschede, The Netherlands

**Keywords:** Imaging (three-dimensional), Phantoms (imaging), Radiofrequency ablation, Thyroid nodule, Ultrasonography

## Abstract

**Background:**

Two-dimensional (2D) ultrasound is well established for thyroid nodule assessment and treatment guidance. However, it is hampered by a limited field of view and observer variability that may lead to inaccurate nodule classification and treatment. To cope with these limitations, we investigated the use of real-time three-dimensional (3D) ultrasound to improve the accuracy of volume estimation and needle placement during radiofrequency ablation. We assess a new 3D matrix transducer for nodule volume estimation and image-guided radiofrequency ablation.

**Methods:**

Thirty thyroid nodule phantoms with thermochromic dye underwent volume estimation and ablation guided by a 2D linear and 3D mechanically-swept array and a 3D matrix transducer.

**Results:**

The 3D matrix nodule volume estimations had a lower median difference with the ground truth (0.4 mL) compared to the standard 2D approach (2.2 mL, *p* < 0.001) and mechanically swept 3D transducer (2.0 mL, *p* = 0.016). The 3D matrix-guided ablation resulted in a similar nodule ablation coverage when compared to 2D-guidance (76.7% *versus* 80.8%, *p* = 0.542). The 3D mechanically swept transducer performed worse (60.1%, *p* = 0.015). However, 3D matrix and 2D guidance ablations lead to a larger ablated volume outside the nodule than 3D mechanically swept (5.1 mL, 4.2 mL (*p* = 0.274), 0.5 mL (*p* < 0.001), respectively). The 3D matrix and mechanically swept approaches were faster with 80 and 72.5 s/mL ablated than 2D with 105.5 s/mL ablated.

**Conclusions:**

The 3D matrix transducer estimates volumes more accurately and can facilitate accurate needle placement while reducing procedure time.

## Key points

- Three-dimensional (3D) matrix ultrasound nodule volume estimations were more accurate than current approaches.
- 3D matrix ultrasound-guided radiofrequency ablation was faster than two-dimensional (2D) while being non-inferior.
- A learning curve is likely needed for 3D matrix transducer use.
- 3D matrix ultrasound has the potential for improved thyroid nodule volume estimation and treatment.

## Background

Thyroid nodules have a prevalence of up to 70% in the adult population [1–3]. Of these nodules, 95% are benign, but depending on their size and number present, they can still cause complaints such as difficulty with swallowing, breathing, and cosmetic negative effects [4–6]. Diagnosis of these nodules is performed primarily with two dimensional (2D) ultrasound (US) using a risk stratification system such as Thyroid Imaging Reporting and Data System (TIRADS) and, if indicated, followed by fine needle aspiration [7, 8]. These procedures are unnecessary in up to 32% of the cases, depending on the TIRADS used [7].

Based on fine needle aspiration results, a therapy may be indicated to achieve symptom relief. According to a European Thyroid Association survey [9], the most performed therapy is the surgical removal of the affected thyroid lobe or thermal ablation for those unwilling or unable to undergo surgery. Minimally invasive thermal ablation, including radiofrequency ablation (RFA), laser ablation, and microwave ablation, may be the first choice for many patients [9–14]. These ablations are highly effective and safe and result in less to no scarring, a lower risk for hypothyroidism, less pain, and less nerve damage as compared with surgery [10, 11, 15–17]. However, parts of the nodule may be left untreated, therefore leading to regrowth which occurs in 5–24.1% of the cases (depending on the follow-up time) with 10–13% of these cases requiring a second intervention [10, 18, 19]. The regrowth is usually identified during follow-up at 3-years post-ablation. However, while advised by the European Thyroid Association, not every hospital has such a long follow-up [9, 13, 18].

Ablative treatments for thyroid nodules are commonly performed under 2D US guidance. Two techniques are commonly used: first, the trans-isthmic approach stabilises the needle and thyroid lobe and helps to avoid ablation within the artificial danger triangle in which the recurrent laryngeal nerve is located [13]; second, the multiple-overlapping-shots technique (MOST), which improves the visibility of the target area during the intervention. MOST entails starting the ablation at the “deepest” point in the nodule, farthest away from the RFA needle entrance [13]. However, 2D US guidance limits the radiologist in not being able to fully monitor in real-time the position of the RFA needle with respect to local vital structures and the nodule treatment area. Increasing the field of view during the intervention would allow to ablate the tissue closer to the edge of the nodule, including that of the danger triangle while avoiding the nearby critical structures.

With the availability of a high-frequency three-dimensional (3D) matrix transducer, it is possible to create real-time 3D US images of the thyroid and nodules. Schlögl et al. [20] showed that their 3D US segmentation volume estimation had a 9.7% deviation as compared to the 2D volume estimation with 26.9%, with the actual volume of the resected thyroid nodules as a reference. Furthermore, 3D US can offer a superior field of view during the intervention and allow for more complete ablation of the nodule while preventing ablating outside of the nodule due to more accurate needle placement. However, to the best of our knowledge, no *in*-*vivo* or *in*-*vitro* studies have reported on the use of 3D US during thyroid ablation interventions.

This study investigates the applicability of a real-time 3D US matrix transducer during volume estimation and RFA intervention of phantom thyroid nodules while comparing it to two conventional US transducers. Thyroid nodule mimicking gel phantoms, with a heat-sensitive ink mixed in, were developed.

## Methods

We performed volume estimation and image guidance of the RFA in these phantoms using three US transducers: (1) real-time 3D US (3D matrix); (2) conventional 2D US; and (3) the older 3D mechanically-swept (3Dms) US. For each of the three transducers, the time of the procedure was tracked and image analysis of the nodule based on colour-change was performed.

### Phantoms

This study was conducted on in-house produced tissue-mimicking thermochromic phantoms. The phantoms were made of polyacrylamide (PAA) gels (Sigma Aldrich, St. Louis, Missouri, USA) with the addition of a thermochromic ink (TMC Hallcrest, Flintshire, UK), which changes colour permanently depending on temperature. The phantoms were created according to a recipe adapted from Negussie et al. [21, 22]. The ingredients are listed in Table 1. The phantoms consist of a body containing a nodule. The phantoms were created in two steps, first the body with a cavity to contain the nodule.

**Table 1:** Ingredients for the body and nodule based on the work of Negussie et al. [21, 22]

Components	Body proportion (%)	Nodule proportion (%)
Deionized water	76.18 (v/v)	74.68 (v/v)
Acrylamide/bisacrylamide	17.5 (v/v)	
Kromagen magenta MB60 concentrate	5.0 (v/v)	
Sodium chloride (NaCl)	0.9 (w/v)	
Ammonium persulfate	0.14 (w/v)	
Tetramethylethylenediamine	0.28 (v/v)	
Silica beads (SiO_2_)	–	1.5 (w/v)

*v/v* Volume concentration volume/volume, *w/v* Weight concentration weight/volume

The phantoms were created in plastic containers of 550 mL. To create the cavity for the nodule, a balloon was filled with water and suspended from the lid of the container; fixated with tape and an iron wire to hold the balloon in a steady position. Thereafter, the container was filled with the PAA solution. The filled containers were placed in a vacuum chamber for 2.5 min to remove the majority of the air bubbles. Subsequently, the containers were closed by placing the lids on the container, submerging the balloons in the PAA solution. The containers were then placed in a refrigerator (4 °C) for a minimum of 3 h to prevent a colour change of the thermochromic ink due to the exothermic reaction of the cross-linking. After cross-linking of the body, the nodule was created by removing the balloons and filling the cavities with a PAA solution with silica beads (SiO_2_) (Sigma Aldrich, St. Louis, Missouri, USA). The addition of silica beads results in a high concentration of scatterers in the nodule, generating speckle and contrast on US. The containers were then placed inside a vacuum chamber for 2.5 min, while being gently rocked from side to side, to remove the majority of the air bubbles in the nodule and as such creating a more tight seal with the body. Thereafter, the lids were put back on, and the containers were kept in a cool room for cross-linking of the nodule and kept refrigerated until used for ablation.

For the initial batch, electrical conduction was measured and the colours of the ink monitored during a temperature bath experiment to verify these with the work by Negussie et al. [21] and Mikhail et al. [22].

### Volume estimation and ablation of the phantoms

**Fig. 1:**
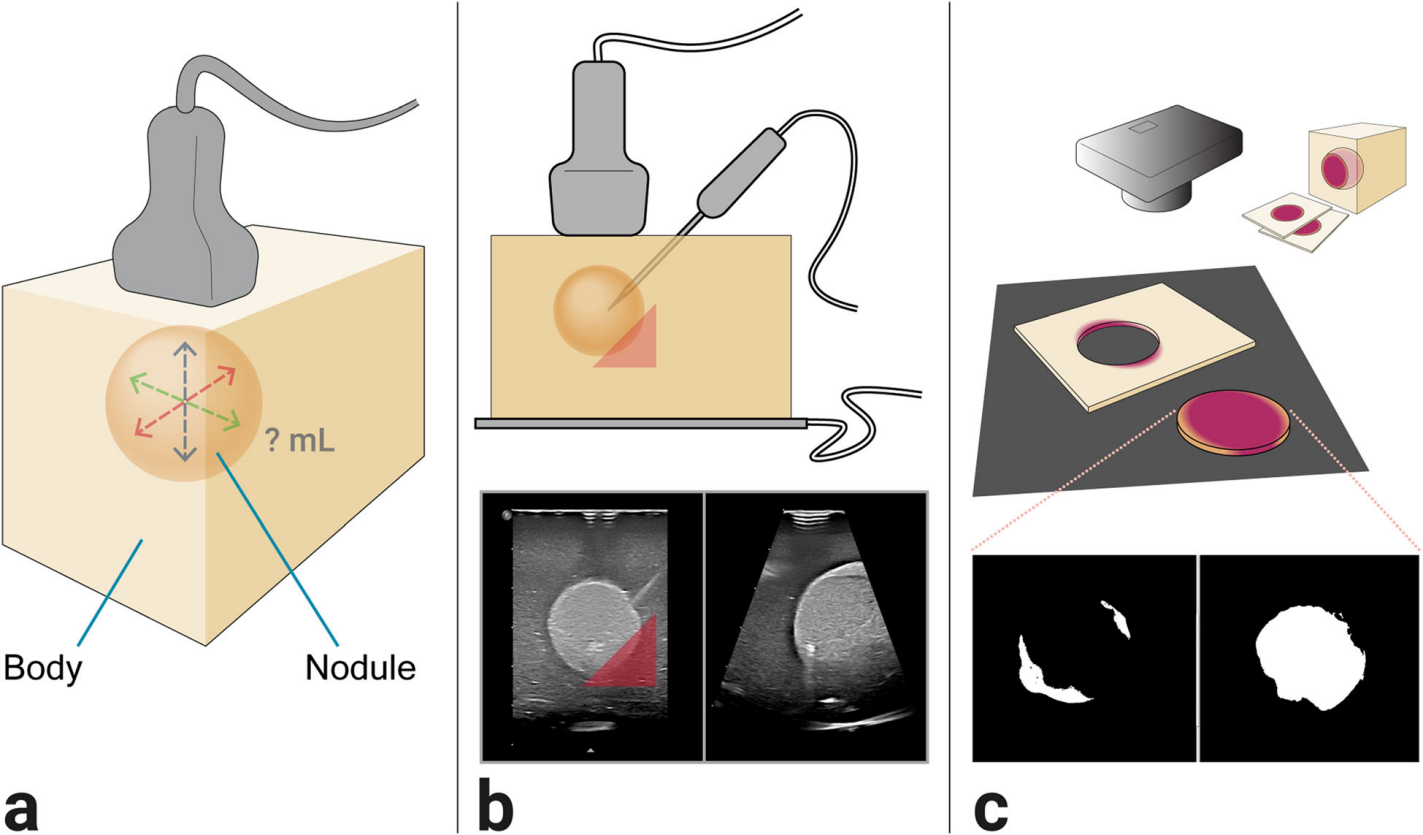
Method depictions. **a** Three-dimensional (3D) view of the phantom with nodule volume in milliliter (mL) being estimated. **b** Side view of the ablation set-up with a grounding pad, radiofrequency ablation needle, and transducer and the red triangle indicating the danger triangle. Beneath that the 3D matrix (real-time 3D transducer) view of a nodule ablation. **c** Slicing, photographing, and pixel-based analysis of the body and nodule after ablation. *2D*: Two-dimensional (conventional 2D transducer); *3D*: Three-dimensional; *3D matrix* Real-time 3D transducer; *3Dms*: 3D mechanically swept transducer;

Both volume estimations and ablations were performed by a clinical expert with 4 years of experience in thyroid nodule RFA. Before ablation was performed, 2D and 3D volume estimations were executed. For 2D US guidance, a linear probe (General Electric Healthcare ML-D, 6-15 MHz) with the LogiQ E9 US system (GE Healthcare, Chicago, Illinois, USA) was used. The manual software calliper measurements of each US system were used to measure in the three principal axis directions (long axis and two orthogonal short axes). Subsequently, volume calculations were performed, based on the volume formula for an ellipsoid ($A\times B\times C\times \frac{\pi }{6}$ as per the protocol widely used in clinics) [23]. For the 3Dms US guidance, a wobbler-probe (7CF2, 2-7 MHz) and the Acuson S3000 (Siemens, München, Germany) with its manual callipers and volume calculation tool were used. The transducer operates at a volume rate of 2–3 volumes/s. For the real-time 3D US guidance, the XL14-3 xMatrix transducer (3–14 MHz), the Epiq Elite (Philips Healthcare, Amsterdam, The Netherlands), and its manual callipers and volume calculation tool were used. This transducer operates at a volume rate of 5–6 volumes/s. The 3D view is based on the Philips *x*-plane function, showing a transversal and axial image of a thyroid lobe at the same time. Ten phantoms were scanned and measured with the 2D transducer (General Electric ML-D 6-15, working frequency 15 MHz), ten with the 3Dms transducer (7CF2, working frequency 7 MHz), and ten with the 3D matrix transducer (XL14-3 xMatrix, working frequency14 MHz); all using their system’s respective thyroid pre-sets. The procedure steps are schematically shown in Fig. 1.

Similarly, the ablation is guided by one of three transducers for each of the ten phantoms. During the procedure, a single needle monopolar radiofrequency electrode (internally liquid-cooled, 7-mm and 360° active zone and sharp needle tip, 18 G) was used in combination with the VIVA radiofrequency system (both from STARmed Co., Ltd., Goyang, South Korea) and a single grounding pad on which the phantom was placed. The ablations were performed with close resemblance to the clinical situation using free-hand guidance and a mimicked transisthmic approach, keeping a danger triangle area (the medio dorsal side) free of ablation, as well as utilising MOST. Ablation was started with 25 W and increased in increments of 5 W at the request of the clinician. A position was considered fully ablated when the ohmic resistance increased rapidly and was at least over 300 Ohms. Time (s) to complete ablation was tracked for each phantom from the moment of needle insertion to needle removal. Since nodule volume can vary, the ablation time is presented per mL as opposed to a median ablation time based on an average nodule size.

### Analysis of the phantoms

The phantoms were manually cut into slices of approximately 5-mm thickness. Slices were cut perpendicular to the needle tracts. The nodule slices were carefully removed from the body slices. This is possible because the nodule was created in the body enclosure, and the nodule only sticks to the enclosing gel without being fused with it, even after heat deposition. This allows the nodules slices to be accurately separated and removed. The front and backside of the body and nodule slices were photographed on a 10 cm × 10 cm black paper, at an angle of 90° and under equal lighting conditions. The paper was used as a dimension reference in the analysis.

For the pixel-based analysis, the MATLAB imaging toolbox (MATLAB R2020a, The MathWorks Inc., Natick, Massachusetts, USA) and its colour thresholder application were used. The nodule areas of each slice were drawn manually to prevent overestimation of the areas, due to the curved edges of those spherical segments and caps. Each area was drawn twice and averaged to reduce the error. The resulting nodular areas of the slices were used to calculate the volume for either a spherical cap or a spherical segment. The summation of the volumes of the slices resulted in the ground truth nodule volume.

For the ablation coverage (nodule) and ablation volume determination (nodule and body), the corresponding values were chosen in the Hue saturation value colour space identifying ablated and non-ablated phantom tissue. The magenta colour indicated the ablated area where the temperature had reached at least 65 °C. The ablation coverages and volumes were calculated per slice and added to determine the totals for the nodule and body. The edge of the ablation zones contains phantom tissue ablated to almost 65 °C. The volume of the edge was determined to further evaluate its relevance.

To compare the frequency of ablated locations outside the nodule per transducer, each phantom slice was visually checked at two locations, *i.e.*, the needle entry point and the danger triangle. Per phantom, if an ablated area was observed in one or more of the slices one point was added to the total for that location and transducer type.

The results—volume estimation (mL), ablation coverage of the nodule, ablated volume outside the nodule (mL), and ablation time (s)—for 2D, 3Dms, and 3D matrix US were assumed to be not normally distributed and thus compared using the Kruskal-Wallis and Dunn’s pairwise comparison tests in SPSS (IBM Corp, version 26.0, released 2019, Armonk, New York, USA). The data were described using the median and interquartile percentiles (25^th^–75^th^ percentile). The significance threshold was set at *p* < 0.05.

## Results

**Fig. 2:**
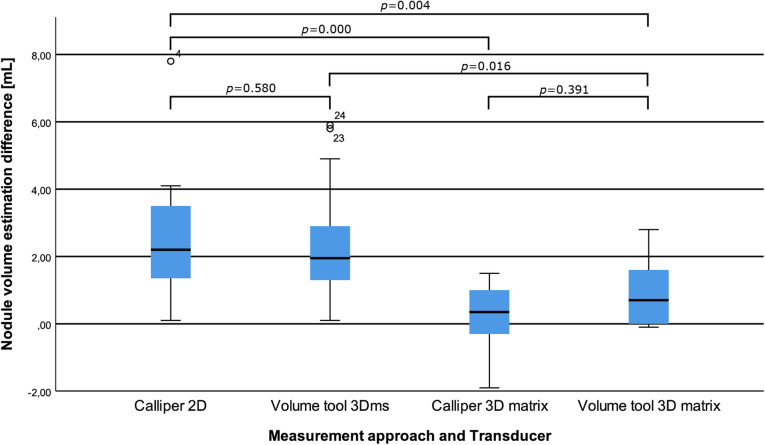
Boxplot of the differences in mL of the volume estimation measurements as compared to the reference value per transducer and measurement approach with corresponding *p* values

Thirty thyroid nodule phantoms mixed with thermochromic ink had their volumes estimated and were ablated under image guidance of one of the three US transducer types. The nodule ground truth volumes had a median size of 13.8 mL with an interquartile percentile range of 13.0–16.2 mL. The 3D matrix transducer showed the least deviation in volume estimation from the ground truth (2.9%) compared to the 2D- and 3Dms-transducers (15.9%, *p* = 0.000 and 14.5%, *p* = 0.016). The volume estimation differences per transducer type can be found in Table 2 and the corresponding boxplot with *p* values in Fig. 2.

**Table 2:** Overview of the four main results shown with medians and interquartile ranges

	Conventional 2D transducer	3D mechanically swept transducer	Real-time 3D transducer
Estimated nodule volume difference	Calliper measurement 2.2 mL (1.3–3.5 mL)	Volume tool 2.0 mL (1.3–2.9 mL)	Calliper measurement 0.4 mL (-0.4–1.1 mL) Volume tool 0.7 mL (-0.0–1.6 mL)
Nodule ablation coverage	80.8% (67.1–92.4%)	60.1% (58.2–71.6%)	76.7% (65.7–85.0%)
Volume ablated outside nodule	4.2 mL (1.2–5.9 mL)	0.5 mL (0.4–1.0 mL)	5.1 mL (4.3–6.8 mL)
Ablation time/mL	105.5 s/mL (69.5–94.5 s/mL)	72.5 s/mL (66.8–77.3 s/mL)	80.0 s/mL (69.5–94.5 s/mL)

*2D* Two-dimensional, *3D* Three-dimensional

**Fig. 3:**
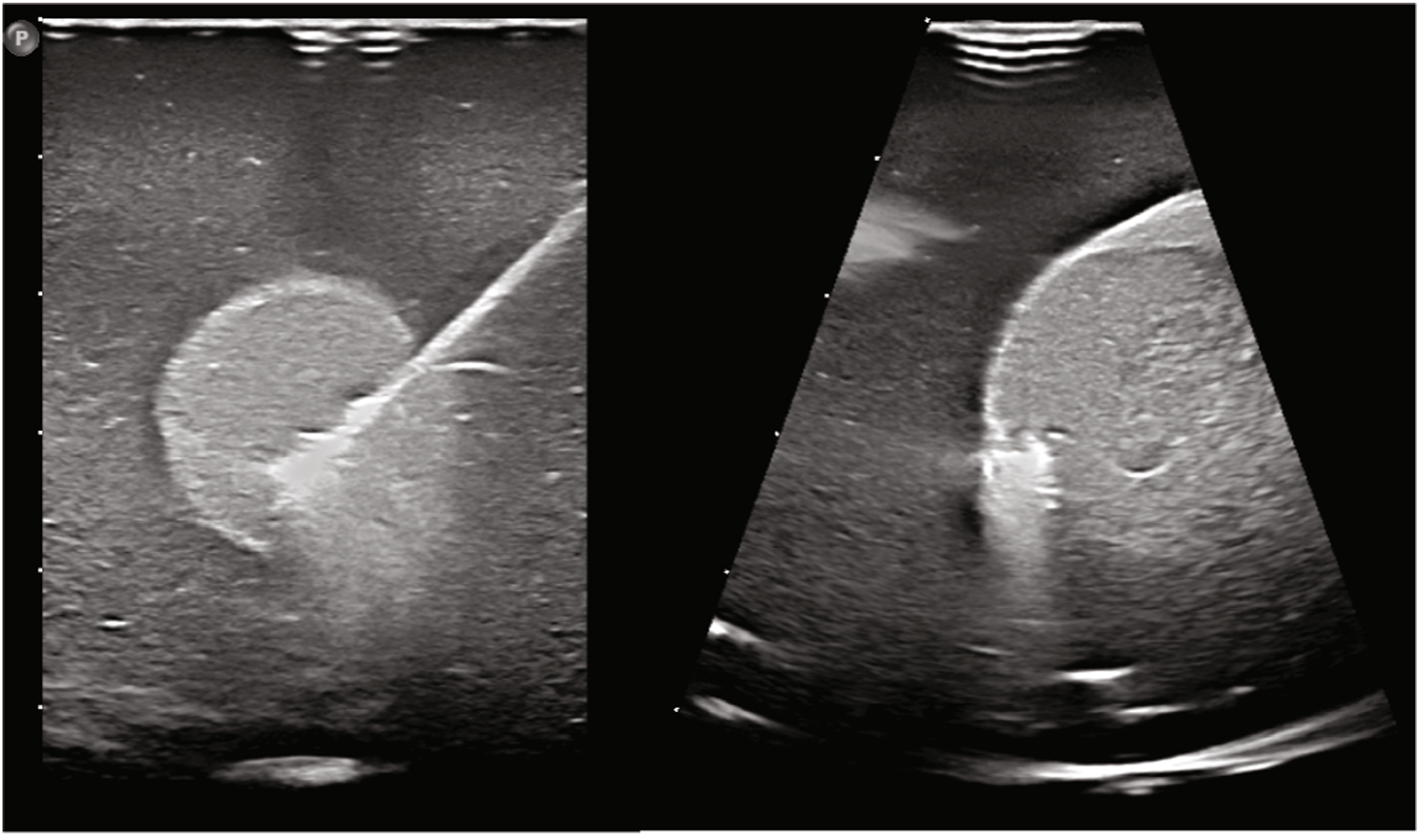
Transversal (left) and sagittal (right) view of the nodule phantom during ablation with the three-dimensional (3D) matrix (real-time) transducer

**Fig. 4:**
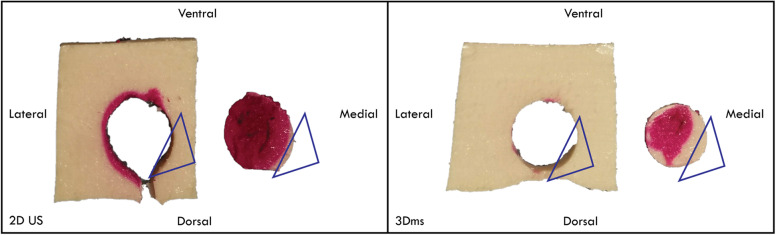
Ablation results using conventional two-dimensional (2D) and three-dimensional mechanically swept (3Dms) transducer guidance. The blue triangle indicates the danger triangle, free from ablation in the nodule and outside the nodule. The magenta colour in the body indicates an overablation outside the nodule

**Fig. 5:**
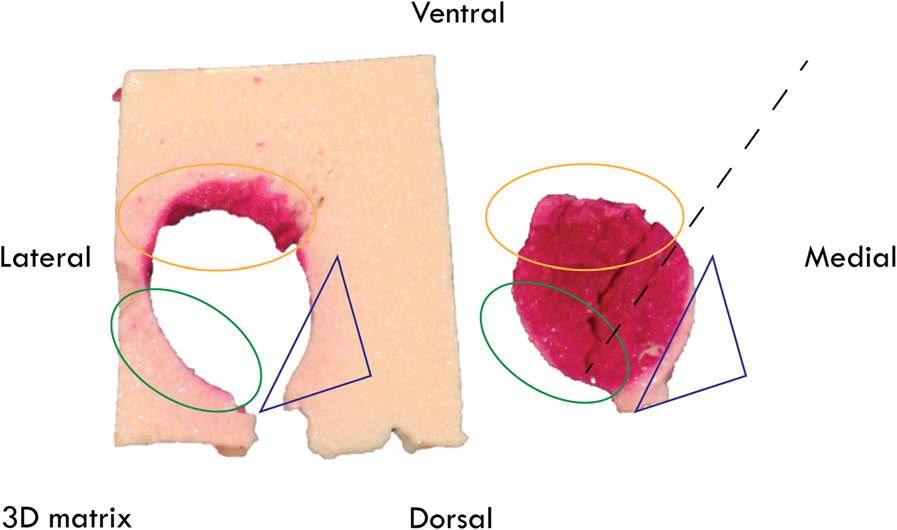
Ablation results using 3D matrix guidance. The dashed line indicates the ablation needle orientation. The green circle indicates an area ablated closely to the edge with minimal ablation outside the target. The blue triangle indicates the danger triangle with zero ablation outside the target. The yellow circle indicates an edge of the nodule fully ablated as well as the area outside of the nodule showing ablation

**Fig. 6:**
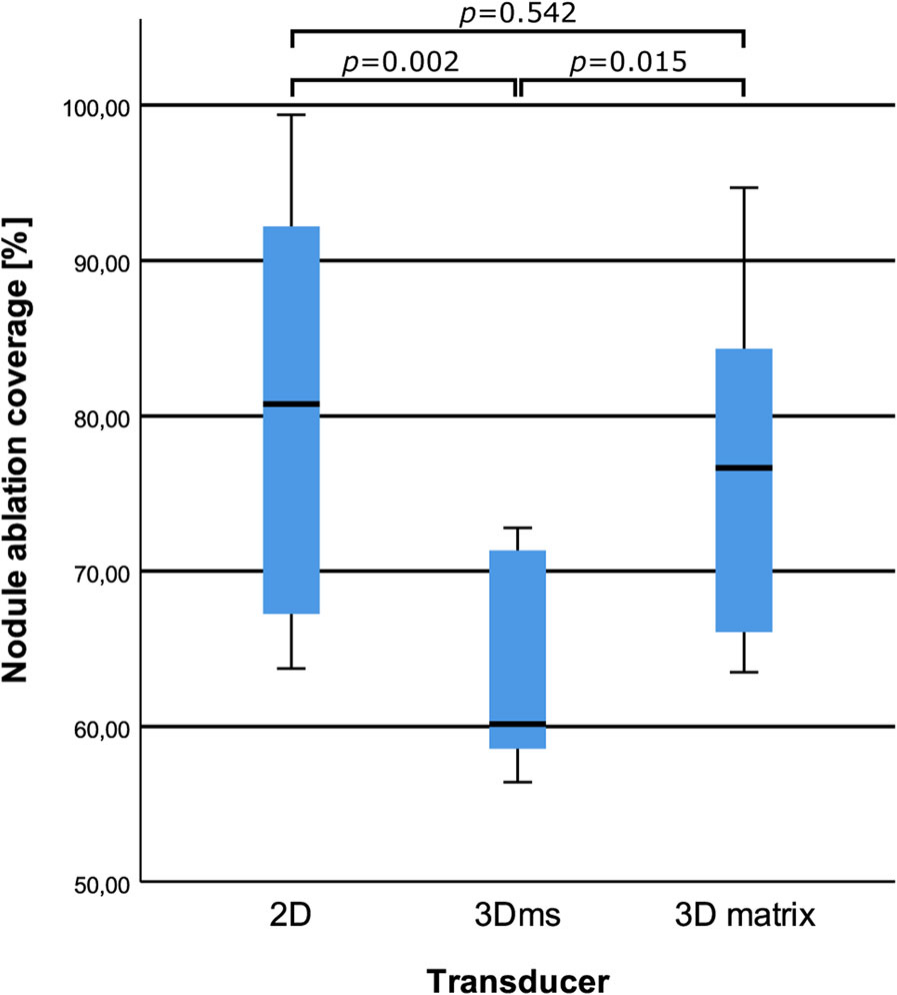
Boxplot of the nodule ablation coverages per transducer with corresponding *p* values

**Fig. 7:**
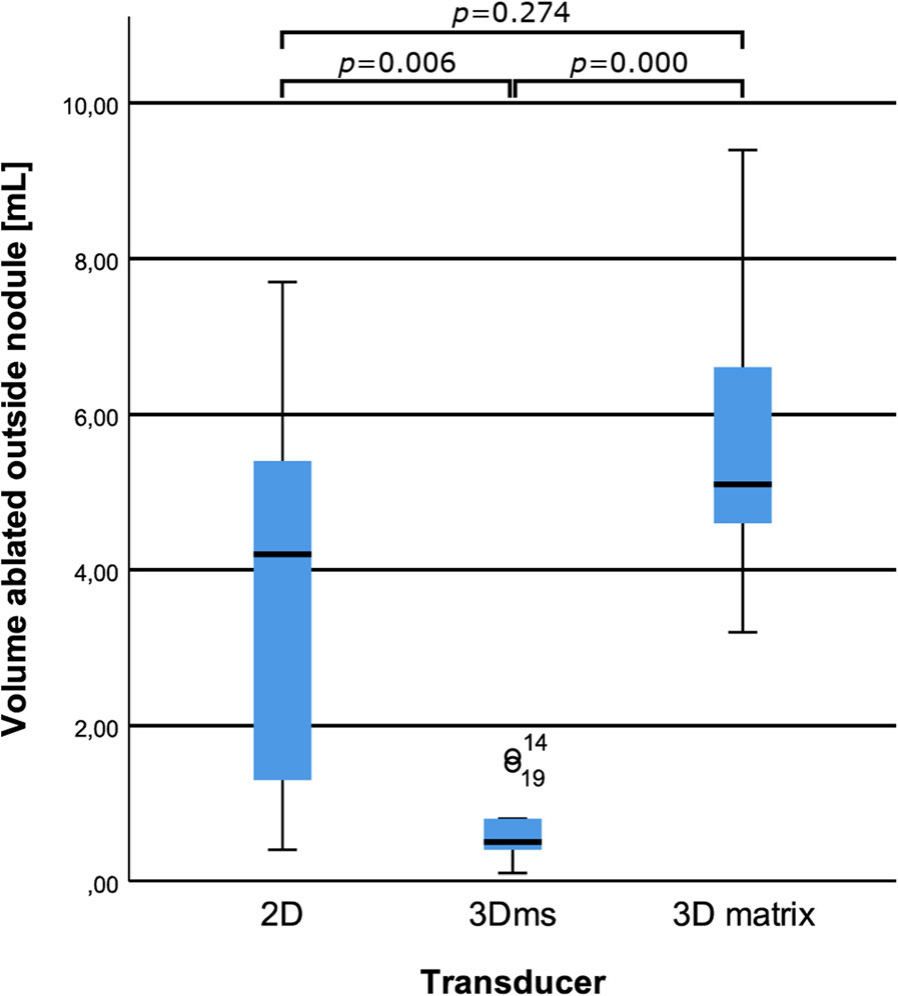
Boxplot of the volume ablated outside the nodule per transducer with corresponding *p* values

The 3D matrix transducer view during RFA of one of the phantoms is shown in Fig. 3. Scattering due to gas bubble formation was observed around the needle tip (*i.e.*, the ablation site). Visual inspection of the slices showed that the areas inside and outside the nodule that were ablated (see Figs. 4 and 5). Furthermore, the 3D matrix and 2D US transducer achieved a non-significant difference in nodule ablation coverages and volumes ablated outside the nodule (76.7%, 5.1 mL and 80.8%, 4.2 mL, *p* = 0.542); the 3Dms transducer was found to be significantly lower (60.1%, 0.5 mL, *p* = 0.015). The nodule ablation coverages and volume ablated outside the nodule can be found in Table 2 and Figs. 6 and 7, respectively. The ablation time per mL can be found in the last row of Table 2 with the following statistical results: 2D *versus* 3Dms *p* = 0.000, 2D *versus* 3D matrix (*p* = 0.011), and 3Dms *versus* 3D matrix (*p* = 0.186). The number of times ablations were observed in the danger triangle and at the area of needle entrance to the nodule for each transducer are shown in Table 3.

**Table 3:** Number of observed ablated locations outside the nodule per transducer (*i.e.*, 10 phantoms)

Transducer type	Needle entrance of nodule	Danger triangle
Conventional 2D	10/10	4/10
3D mechanically swept	8/10	1/10
Real-time 3D	10/10	2/10

*2D* Two-dimensional, *3D* Three-dimensional

Furthermore, electrical conduction and thermochromic ink colour change matched with other studies [21, 22]. Additionally, the edge of ablation, *i*.*e*., the zone showing discolouration though not having reached 65 °C, is 0.12-mm-thick at maximum which translates to a volume of 0.35 mL on average.

To acquire experience ablating these phantoms, the 2D and 3Dms ablations were performed before in a test run with 20 phantoms; their respective ablation coverages for the test run were 67.4% (61.1–72.0%) and 52.2 (44.0–59.8%). Their respective volumes ablated outside the nodule were 1.0 mL (0.6–1.4 mL) and 0.8 mL (0.3–1.1 mL).

## Discussion

We investigated in a phantom study whether the volume estimation derived by using a 3D matrix transducer can represent the actual volume more closely than those derived with 2D and 3Dms transducers and whether the 3D view facilitates more accurate needle placement that aids radiofrequency ablation. The 3D matrix transducer represented the actual volume the closest. Whereas during RFA, the 3D matrix guidance differed not significantly from the 2D guidance.

Currently, volume and size estimations are performed by calliper measurements. Earlier studies have shown that calliper measurements may be prone to inter and intra-observer variability (13–17% and 814%, respectively) [24, 25] and result in more inaccurate thyroid volume measurements (17.2% larger deviation) [20]. Reducing this variability and deviations in US volume estimations is, therefore, desirable [24]. This study showed that it is possible to apply 3D US to reduce this deviation from the actual thyroid volume, even though these phantom nodules are sphere-like and thereby facilitate volume estimation accuracy.

Three-dimensional US can even perform at the level of computed tomography or magnetic resonance imaging volume estimations, as was studied by Baek et al. [26]. This high level of accuracy is beneficial in the clinical setting. Because thyroid nodules have varying shapes, especially during follow-up after ablation, they are more difficult to quantify (*e.g.*, using the ellipsoid volume formula). The accuracy and speed of 3D volume estimation may be improved by various approaches such as contrast-enhanced US and a series of computer vision approaches. Contrast-enhanced US has shown to be promising in determining the size of the nodule before ablation and after the intervention to improve the intraobserver and interobserver reproducibility as compared to standard 2D US (85% *versus* 27% and 95–96% *versus* 21–23%) [27]. These computer vision approaches can be for instance autosegmentation with contour and shape-based methods, that allow to describe more complex geometrics. Further research is required to develop such a method in an *in vivo* setting [28].

For the clinical expert, it was the first time both using the 3D matrix and 3Dms transducer. For the 3D matrix transducer, this suggests that from the start of the procedure the clinical expert was more intuitively able to position the needle, making the procedure faster. Bubble artefacts were encountered during the 3D matrix-guided ablations similarly as seen with 2D US. The extra scanning plane does offer an extra view on how this bubble “cloud” grows and thus may serve as an extra guidance on how far the ablation zone is reaching. However, further research has to be performed to validate this. Comparing the RFA results with our earlier test run wherein twenty 2D and 3Dms guided ablations were performed, an increase in ablation coverage was observed for both 2D and 3Dms ablations. This may indicate a learning curve in ablating these phantoms, thus improved results in future runs with the 3D matrix transducer may be expected.

Nevertheless, 3D matrix guidance resulted in a non-significant difference of ablated volume outside the nodule as with the 2D-guided ablation. This indicates that estimation of the range of the ablation in the current 2D and 3D view, by the clinical expert, is challenging. The *x*-plane view of the 3D matrix transducer adds a scanning plane which will be similarly challenging and perhaps even more, due to having two scanning planes at the same time is new for clinicians. This inexperience with the 3D matrix transducer may also have led to the larger ablated volume outside the nodule while having a lower nodule ablation coverage as compared to the 2D-guided ablation. Further research should focus on full nodule ablation, especially the periphery of the nodule, as it is important in controlling regrowth according to Sim et al. [18]. Therefore, training with the 3D matrix transducer and an ablation planning and guiding tool may be beneficial in reducing volume ablated outside the nodule, while achieving a full ablation. A comparable planning and guidance tool has been developed for and is currently tested in the liver and may be translated to the thyroid as well [29]. The first step has been taken already with a guidance tool for RFA needles in the treatment of thyroid nodules, showing the feasibility and use of such tools by reducing the number of punctures required and in the future allowing closer ablation to nearby critical structures [30].

Two important things are left to note concerning the ablations. First, the ablated volume outside the nodule at the nodule entrance location may likely be attributed to another factor, making ablation zone estimation even more challenging. Dobnig and Amrein [31] showed in their study the characteristic ablation pattern for monopolar RFA needles, which described an elongated ablation pattern alongside the covered part of the needle upwards to the handle (*i.e.*, toward the needle entrance location). Secondly, the matrix transducer operated at a volume rate of 5–6 volumes/s which was sufficient to perform an intervention, in this stable situation. The 3 volumes/s volume rate achieved with the 3Dms transducer made it difficult to work with. This caused the expert to work with more caution than normal, resulting in the lower ablation coverage which inherently leads to a lower ablated volume outside the nodule.

We have produced the phantoms to mimic the thyroid as closely as possible, while not making it unnecessarily complex. Almost all characteristics of PAA gels are comparable to those of the thyroid, only the acoustic attenuation is low [32]. For this superficial structure, it could have improved the overall visibility of the nodule; however, due to the already distinguishable boundary between body and nodule, this lack of attenuation was deemed to have minimal impact on the study outcome. The high contrast of the boundary of the nodule was a clear indicator for the expert to follow; this might have helped during ablation affecting the ablation coverage and ablated volume outside the nodule in both a positive and a negative way, respectively. To limit the phantom complexity for this study, two details were omitted: the heat sink effect and surrounding critical structures. First, Nolte et al. have shown, in their unpublished observations “Study of flow effects on temperature-controlled radio-frequency ablation using phantom experiments and forward simulations,” that a heat-sink effect does reduce the volume in these kinds of phantoms. Thus for this study that has likely resulted in more volume ablated outside the nodule, however, this has happened for all phantoms and therefore does not affect the comparison between the transducers. Second, the lack of surrounding critical structures to be used for orientation make this simulation less realistic. However, with the addition of an artificial danger-triangle, utilising the transisthmic approach and MOST, this study closely approached reality. This simulation was equal between the three US transducers. The purpose of this study was to find differences between the transducers, and with these in-house produced phantoms we managed to build a controlled environment to study this. In the future, phantoms with heterogenous echogenicity and varying shapes of the nodules will be used.

The analysis was performed in a standardised way to obtain comparable results. Although minimised, some manual errors were unavoidable. First, volume estimations are based on masks drawn by hand, resulting in a manual error, which was minimised by averaging the two masks of the two slices with the same position. Secondly, the Hue saturation values have been manually selected for each photographed batch individually. Although this is a manual selection, the edge of the ablation zone wherein the colour changes is small. Thus the impact of manual selection is limited. Third and lastly, the ablation zone inside the nodule was assumed to have a sphere-like shape for its volume calculations, since upon visual examination the extent of any bulbous areas of the ablation zone was small. Therefore, this assumption is not likely to have over or underestimated the actual ablation coverage.

In the future, further training and research with advanced heterogeneous phantoms is required, to see if a reduction in volume ablated outside the nodule can reach clinically acceptable levels or that ablation guiding tools should be developed. Furthermore, with the increased accuracy in volume estimation for the matrix transducer, a study can be performed into replacing the longest-axis cutoff points in the TIRADS protocols with volume cutoff points, potentially reducing the number of unnecessary biopsies. Considering the follow-up, further research has to be performed wherein the 3D US scans are utilised in combination with an automatic nodule segmentation method. The resulting volume estimation for ablated and unablated nodular tissue, with often irregular shapes, can then be performed with higher accuracy improving the recognition of regrowth.

In conclusion, we have shown that 3D matrix transducer guidance improved nodule volume estimation accuracy, and reduced RFA procedure time. Furthermore, it is noninferior and nonsuperior to 2D guidance when comparing their nodule ablation coverages and ablated volume outside the nodules. The 3D matrix technology allows for a dual view on the needle position with respect to the nodule boundary, aiding in accurate needle placement, although further research and development is required.

## Data Availability

The datasets used and/or analysed during the current study are available from the corresponding author on reasonable request.

## Abbreviations

2D: Two-dimensional (conventional 2D transducer)
3D matrix: Real-time 3D transducer
3D: Three-dimensional
3Dms: 3D mechanically swept transducer
MOST: Multiple-overlapping-shots technique
PAA: Polyacrylamide
RFA: Radiofrequency ablation
TIRADS: Thyroid imaging reporting and data system
US: Ultrasound
